# Effectiveness of Chinese Herbal Medicine Combined with Antibiotics for Extensively Drug-Resistant Enterobacteria and Nonfermentative Bacteria Infection: Real-Life Experience in a Retrospective Cohort

**DOI:** 10.1155/2017/2897045

**Published:** 2017-10-11

**Authors:** Yangping Cai, Qing Zhang, Yuefeng Fu, Li Li, Ning Zhao, Aiping Lu, Qingquan Liu, Miao Jiang

**Affiliations:** ^1^Beijing University of Chinese Medicine Affiliated Dongzhimen Hospital, Beijing 100700, China; ^2^Institute of Basic Research on Clinical Medicine, China Academy of Chinese Medical Sciences, Beijing 100700, China; ^3^School of Chinese Medicine, Hong Kong Baptist University, Kowloon Tong 999077, Hong Kong; ^4^Beijing Hospital of TCM, Beijing 100010, China

## Abstract

Chinese herbal medicines (CHMs) have been successfully used in the treatment of infectious diseases, yet the effectiveness of CHMs for extensively drug-resistant enterobacteria (XDRE) infection remains unclear. Herein we developed a retrospective multicenter study including 766 patients with XDRE and nonfermentative bacteria (NFB) infection to investigate the effectiveness of CHMs combined with antibiotics in the treatment of XDRE infections in clinical daily practice in a cohort of patients and compared the regular antibiotics monotherapy. After 14-day treatment, the 547 patients accepted CHMs combined with antibiotics therapy indicating a more desirable effectiveness compared to the 219 patients treated with antibiotics monotherapy. The primary evaluation indexes included white blood cell count (WBC) and percentage of neutrophil (N%) in blood test. Secondary evaluation indexes consisted of body temperature, breath, heart rate, platelets, hemoglobin, red blood cell, albumin, creatinine, glucose, and 28-day survival rates. Briefly speaking, in our experience, CHMs combined with antibiotics therapy achieved more desirable effectiveness in treating XDRE infections compared with antibiotics monotherapy, and CHMs might be a potential huge resource in the field of XDRE infection management and enlighten the new antibiotics research and development. This trial is registered with ChiCTR-ORC-17011760.

## 1. Introduction

Beginning with the discovery of penicillin, antibiotics have saved millions of patients in the world and brought a revolution in the field of infectious diseases. However, due to the overuse of antibiotics, such as carbapenem, a crisis has been posed here that many antibiotics are no longer effective against even the simple infections worldwide [1] and antimicrobial resistance has been regarded as one of the most serious global public health threats in this century [2].

More and more types of bacteria are no longer susceptible to the common antibiotics treatment. Extensively drug-resistant enterobacteria (XDRE) are a type of Gram-negative bacteria with resistance to multiple antibiotics; generally, these nonfermentative Gram-negative bacteria are natural or acquired drug-resistant to 5 to 7 types of antibiotics, including* Pseudomonas aeruginosa* (PsAr),* Acinetobacter baumannii* (AB),* Klebsiella pneumoniae* (KP), and* Escherichia coli* (*E. coli*). A definition of “extensive drug resistance” designates resistance of a pathogen to all but 1 or 2 classes of antimicrobial agents, among those that are available at the time of use [3]; thus these XDREs usually pose challenges in clinical practice.

Infections caused by such bacteria often result in an increased amount of hospitalization, more treatment failures, higher morbidity and mortality, and prolonged hospitalization [4], especially among the patients in the intensive care units (ICU) and with other serious diseases. The US Center for Disease Control and Prevention (CDC) conservatively estimated that more than two million people every year in the US are affected with antibiotic-resistant infections, with at least 23,000 dying as a result [5]. In Europe, the number of infections and deaths caused by the most frequent multidrug-resistant bacteria (*S. aureus*,* E. coli*,* Enterococcus faecium*,* Streptococcus pneumoniae*,* KP,* and* PsAr*) was estimated at 400,000 and 25,000, respectively, in the year of 2007 [6]. Thus, not only do these bacteria pose a serious threat to global public health, but also they cause a significant burden to healthcare systems.

Consequently, now there is an urgent need to develop new and effective antibiotics to avoid returning to the “preantibiotic era.” Yet there has been a steady decline in the discovery of new and effective antibiotics for diverse reasons [7], such as increased costs, lack of adequate support from the government, poor returns on investment, regulatory hurdles, and pharmaceutical companies that have simply abandoned the antibacterial field.

Chinese herbal medicines (CHMs), which have been used effectively in various infectious diseases in China for thousands of years, are regarded as a huge resource for new drug discovery [8]. Some studies have provided potential evidences that CHMs can be effectively used in treating infections caused by XDRE [9–11]. However, it is difficult to explore the exact data on the overuse of antibiotics [12] and epidemiology of XDRE in healthcare in China [13], much less the reports on clinical effectiveness of CHMs in treating XDRE infections, which should be the basic step in the procedure of new drug development for treating XDRE infections based on CHM.

Therefore, we developed this multicenter study to investigate the effectiveness of CHMs combined antibiotics in the treatment of XDRE infections in clinical daily practice in a cohort of patients and compared the regular antibiotics monotherapy.

## 2. Patients and Methods

This study includes all consecutive patients who met the inclusive criteria at 5 hospitals in China (Beijing Friendship Hospital, Beijing University of Chinese Medicine Dongzhimen Hospital, First Teaching Hospital of Tianjin University of TCM, Henan Province Hospital of TCM, and Shandong Province Hospital of TCM) between January 2010 and December 2013.

All patients were diagnosed with infection by at least one type of listed bacteria:* Pseudomonas aeruginosa* (PsAr),* Acinetobacter baumannii* (AB),* Klebsiella pneumoniae* (KP),* Escherichia coli* (*E. coli*) by blood, phlegm, urine, or wound secretion sample culture and drug sensitive test. The terms “extensive drug resistance” designates resistance of a pathogen to all but 1 or 2 classes of antimicrobial agents, among those that are available at the time of use of the definition and in most parts of the world and that are regarded as potentially effective against the respective pathogen [3].

The infected location could be in pulmonary or urinary system or postoperative wound infection. Combined infection with 2 or 3 kinds of bacteria was also included. Patients would be excluded if they were diagnosed with any psychotic disorders, or they had been included in any other clinical trials, or they were in gestational period or lactation women, or they were allergic to any CHMs, or their complete records could not be acquired. Each patient signed written informed consent before enrollment.

The case records of all patients were reviewed and retrospective data extracted systematically by using a standardized clinical report platform in each hospital. The demography information included name, age, body height, body weight, gender, nationality, appetite, disposition, and chronic diseases. The date of a positive result of any sample culture (blood, phlegm, urine, and wound secretion) with any of the four types of bacteria was set as the first day of this study; clinical data including vital sign and experimental examination such as hemoglobin (HBG), red blood cell (RBC), white blood cell count (WBC), percentage of neutrophil (N%), platelet (PLT), blood glucose (GLU), creatinine (Cr), blood urea nitrogen (BUN), total bilirubin (TBIL), and total bilirubin (ALB) were recorded and analyzed on the 3rd, 5th, 7th, and 14th day after treatment, respectively. The 28-day survival rates in the two groups were also calculated.

There were altogether 766 cases included in this study, and they were classified into traditional Chinese medicine treatment group (TCM) and antibiotics treatment group (Control group) according to the therapeutic remedy. The 547 patients in TCM group were treated with TCM herbal formula combined with antibiotics; 219 patients in Control group accepted antibiotics monotherapy. Since the infections were all caused by extensively drug-resistant bacteria and some patients were even infected with more than one kind of bacteria, treatment remedy was decided completely by physicians.

The antibiotics used in this study included carbapenems, cephalosporin, and aminoglycosides; drug combination was not restricted. The dosage of antibiotic was decided according to the dispensatory and creatinine clearance rate.

The 547 patients in TCM group accepted Chinese medicine treatment combined with antibiotics therapy. The applied Chinese medicine included decoction, Chinese patent drugs, and Chinese medicine parenteral solutions; the principle of treatment was clearing heat and detoxifying, reinforcing Qi, and activating blood. The frequently used herbal medicines consisted of* Flos Lonicerae* (Jin yin hua),* Radix Angelicae Sinensis* (Dang gui),* Radix Astragali seu Hedysari* (Huang qi). In some prescription, there were also* Radix Paeoniae Rubra* (Chi shao),* Radix Rehmanniae Recens* (Sheng di huang),* Fructus Gardeniae* (Zhi zi), and so on. The prescription and treatment course were all decided by physicians.

All patients were observed for 14 days and followed up for another 14 days.

The response to treatment was assessed primarily by changes of routine analysis of blood, mainly by WBC and N% level; secondarily by blood biochemical examination and vital signs (body temperature, breath, and heat rate).

All data was analyzed by SPSS21.0 software. All data except demographic data were expressed as mean ± SD and were analyzed by means of Student's *t*-test or rank test. Demographic data was analyzed by means of Chi-square test. A *P* value of less than 0.05 was considered statistically significant.

The trial was registered at Chinese Clinical Trial Registry (ChiCTR, http://www.chictr.org.cn) with the clinical trial registration number ChiCTR-ORC-17011760.

## 3. Results

### 3.1. Clinical Characteristics

The clinical characteristics of the included patients are summarized in Tables 1 and 2.  Table 1 presents the distribution of infected bacteria type and infected location of the patient; most patients were infected with 1 type of XDRE; minority of them were diagnosed with infections by 2 or 3 types of XDRE combination.

There were 190 cases being diagnosed with PsAr infection, including 156 PI, 35 USI, and 19 PWI; 168 with AB infection, including 157 PI, 34 USI, and 11 PWI; 136 with KP infection, including 116 PI, 18 USI, and 9 PWI; 287 with* E. coli* infection, including 86 PI, 177 USI, and 42 PWI. Some cases were diagnosed with infection by 2 or 3 kinds of bacteria simultaneously; the distribution of infection sorted by bacteria category was listed in Table 1. Since there might be more than 1 infected location in one patient, the total infected locations were not equal to the patient numbers.

The clinical characteristics of the included patients in both groups are summarized in Table 2. Five hundred and forty-seven patients were included in TCM group (male versus female, 293/254) and 219 were in Control group (male versus female, 124/95). The median age was 68 years old (range 18–101 years) and 63 (range 18–98 years) in TCM and Control groups, respectively. There were no significant differences in gender and age distribution between the two groups (*P* > 0.05).

There were also no significant differences in temperature, heart rate, WBC, N%, RBC, PLT, Cr, GLU, and ALB between the two groups (*P* > 0.05), yet concerning breath, HGB, and TBIL there were significant differences between the two groups (*P* < 0.05); the values of the 3 items in TCM group were all lower than in Control group.

### 3.2. Response to Treatment

**Figure 1:**
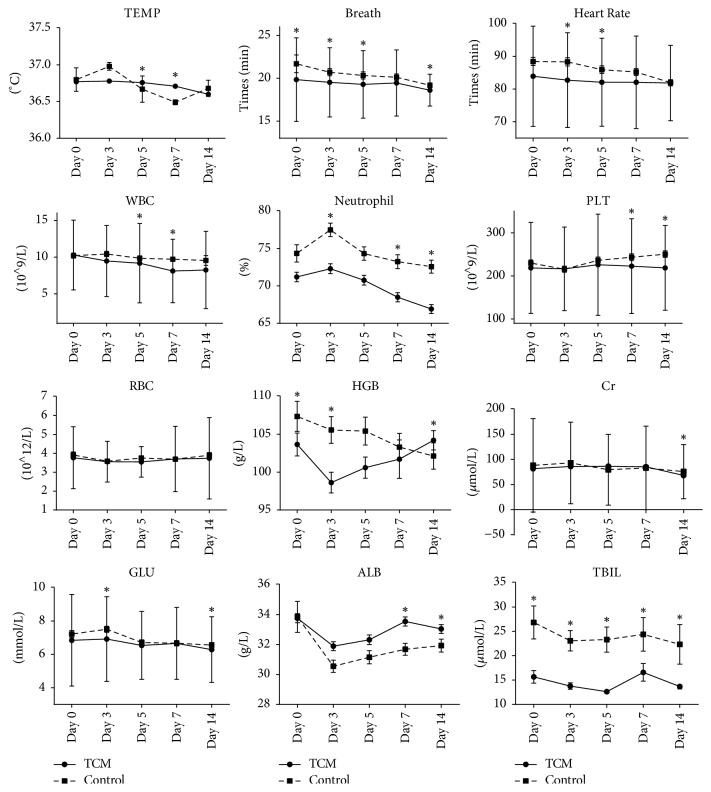
Change of each index before and after treatment in TCM group and Control group.* Note*. The curves show the change of each index before and after treatment in TCM group and Control group from before treatment (Day 0) to the 3rd, 5th, 7th, and 14th days after treatment. The curves were described by the means with error bars (SME). The full line presents the changing in TCM group while the dashed line stands for Control group. An asterisk indicates a significant difference (*P* < 0.05) between the two groups.

Response to treatment is summarized in Table 3 and shown in Figure 1. Primary effectiveness was assessed mainly by WBC and percentage of neutrophil (N%) in blood test for those two items reflects the severity of infection. After 3 days of therapy, WBC began to decrease in both groups, but there was no significant difference between the two groups; on the 5th and the 7th day, there were significant differences between groups: WBC in TCM group decreased rapidly compared to Control group; on the 14th day, the WBC level in TCM was still lower than in Control group, yet no significant difference was detected.

Concerning N%, the changes of the curves in both groups presented a similar trend that increased in the 3rd day, then kept decreasing. There were significant differences in N% between the 2 groups in the 3rd, 7th, and 14th days after the therapy; in TCM group, the N% was significantly lower than in Control group.

Temperature fluctuated more violently in Control group than in TCM group; the temperature increased in the 3rd day and then decreased in the 5th and 7th day, after that it increased in the 14th day. In TCM group, temperature kept decreasing steadily comparatively. HR and breath rate both decreased steadily in the two groups and were lower in TCM group than in Control group.

PLT decreased after 3 days of therapy and then increased on the 5th day in both groups, then kept increasing in Control group, yet decreasing in TCM group. On the 7th and 14th day, PLT were significantly lower in TCM group than in Control group.

HGB kept decreasing in Control group: in TCM group, HGB firstly decreased after 3 days of therapy and then kept increasing. HGB was lower in TCM group in the 3rd, 5th, and 7th day than in Control group and yet was significantly higher in TCM group than in Control group on the 14th day after therapy.

There was no strong change with RBC item after therapy in both groups.

Concerning the safety items ALB, Cr, GLU, and TBIL, they showed similar changing trends in both groups. After 14 days of treatment, ALB was significantly higher, Cr and GLU were significantly lower in TCM group compared with in Control group, and the change curves were relatively steady in TCM group.

After 28 days of treatment, there were 472 and 179 patients who survived in TCM and Control group, respectively. The 28-day survival rates were 86.29% and 81.74% in the two groups, with RR = 1.406 [95% CI = 0.924–2.1420], *P* = 0.071 > 0.05. Although there was no statistical significance between the two groups concerning the 28-day survival rate, the RR value indicated a weak correlation between the exposure factor (CHMs) and the survival rate.

### 3.3. Adverse Drug Reactions

No relevant adverse drug reactions (ADRs) were reported by the included patients in both groups in this study.

## 4. Discussion

To our knowledge, this is the first report on CHMs treating XDRE infections based on a real-world experience. It was indicated that, compared with antibiotics monotherapy, CHMs combined with antibiotics therapy could achieve more desirable outcomes in improving the temperature, WBC, and N% levels, and so forth. WBC and N% are usually used as first-line index to evaluate the violence of inflammation. Bacterial infection can cause inflammation reaction; the systemic inflammatory response syndrome (SIRS) [14] is assessed by the presence of any of the two items within the four indexes: temperature (>38°C or <36°C); breath (>20 times per minute); HR (>90 times per minute); WBC (>12 × 109/L or <4 × 10*e*/L) or neutrophilic granulocyte band form (>10%). Therefore, we selected these four indexes in our study to assess the inflammation as primary evaluation of effectiveness. We found that, in TCM group, temperature, HR, WBC, and N% all presented more steadily and effectively a decrease than in Control group, which indicated better effectiveness of CHMs combined with antibiotics monotherapy.

A severe infection might cause sepsis with secundum dysfunction of multiple organs and bone marrow depression. Thus to monitor the function of internal organs is also very important in the treatment procedure. Cr level is a sign in reflecting the kidney function; TBIL and ALB are indicators of liver function; HGB, RBC, and PLT can reflect the bone marrow depression level; GLU denotes the metabolic status and stress reaction to some extent. So we also included these indexes as secondary evaluation on the effectiveness. The results showed that, in TCM group, both Cr and GLU decreased, and HGB and ALB increased more significantly, compared with in Control group; PLT curve was kept more steadily than in Control group; all the manifestation indicated a better outcome in TCM group concerning the internal organs function protection.

The 28-day survival rate is another frequently used index in such kind of clinical study. In this study, there was no statistical significance between the two groups concerning this item, yet the RR value (1.406) suggested a weak correlation between the exposure factor (CHMs) and the survival rate.

XDRE infection often constitutes a therapeutic challenge. Besides the direct damage from the pathogenic microorganism and the toxins, resistance mechanism disorders play essential roles in the development of XDRE infection. Although there are still scarce antibiotics available, the effectiveness and safety are not desirable and stable; one possible reason is that antibiotics monotherapy might inhibit the reproduction of T and B cells in the ultra-early stage after infection thus to impact the immunologic function. CHMs have been widely used in the treatment of infectious diseases successfully for thousands of years; some CHMs or the formulae were detected to possess antimicrobial activities both experimentally and clinically [15–17]. Due to the multicomponents and multitargets characteristics, CHMs might achieve better effectiveness in treating XDRE infection compared with regular antibiotics monotherapy.

However, the lack of consensus on the effectiveness evaluation of CHM products in treating XDRE infections based on real-world data has limited the new drug development based on CHMs resources; this study was developed as a preliminary and potentially beneficial study.

Although the Chinese medicines used in this study were not unified, there was a basic prescription which was applied in almost all prescriptions, consisting of 3 herbs,* Flos Lonicerae* (Jin yin hua),* Radix Angelicae Sinensis* (Dang gui), and* Radix Astragali seu Hedysari* (Huang qi). These 3 herbs possess different functions based on traditional Chinese medicine (TCM) theory.

Jin yin hua is one of the most common CHMs used for clearing heat and detoxifying; this herb possesses a variety of bioactive effects, such as antibacterial [18], antipyretic [19], anti-inflammatory [20], and antiviral properties [21] and liver protection [22]. Jin yin hua contains more than 140 compounds, including flavonoids, iridoids, organic acids, and saponins, such as chlorogenic acid, luteolin, loganin, and loniceroside A [23]. Forsythoside A has strong antioxidant, antibacterial, and antiviral activities [24]. Jin yin hua is a potent agent for treating various bacteria [25].

Huang qi is another most popular and important Chinese herb in China, with the function of enhancing qi. Qi has lots of essential functions, including defensing the infection with bacteria or virus. More than 100 compounds have been isolated and identified from Huang qi, including flavonoids, saponins, polysaccharides, and amino acids, and various biological activities of the compounds have been reported [26], such as anti-inflammatory [27], antiviral [28], immunomodulating [29], antihyperglycemic [30], and antioxidant [31] activities.

Dang gui is another widely used herb in China, which contains more than 80 composite formulae. The major chemical components identified in Dang gui, such as phthalides, organic acids and their esters, and polysaccharides, are related to the bioactivities and pharmacological properties of Dang gui [32]. Dang gui has been reported to be able to increase the resistance of the rats against PsAr lung infection in a rat mode mimicking cystic fibrosis [33], and the potential mechanism might be its stimulation of the immune system. This herbal medicine is usually combined with other herbs in the clinic, such as Huang qi [34]. The in vitro or in vivo bioactive constituents of herb pairs may differ from those of the single herbs.

Dang gui and Huang qi are often prescribed together, the two herbs compose a formula, Dangguibuxue decoction, which is the most frequently used TCM formula with a function of tonifying qi and enriching blood [35, 36]. This remedy has been used for various diseases for more than 800 years in China [37] and shows broad-spectrum bioactivities such as enhancing bone regeneration [38], attenuating pulmonary fibrosis [39], stimulating proliferation of T-lymphocytes proliferation [40], and alleviating renal damage and diabetic nephropathy [41]. According to TCM theory, to enhance qi and blood can help the body clear the evil qi, which often indicates the bacteria or virus.

Based on our previous study, it has been reported that a CHM formula consisting of Jin yin hua, Huang qi, and Dang gui, named as Qiguiyin formula, can moderately downregulate the lymphocyte proliferation in rats with multidrug-resistant* Pseudomonas aeruginosa* infection and can increase the release of proinflammatory cytokines in early inflammatory response. Compared with the excessive inhibition of immune response by antibiotics monotherapy, Qiguiyin formula could better balance the immune disorders caused by infection and remove the bacteria and toxins. Then as time goes by, this formula can decrease the release of proinflammatory cytokines rapidly, so as to maintain the normal inflammatory response level and prevent the damage caused by prolonged inflammatory reaction [42, 43]. These mechanisms ensured the desirable effectiveness in our clinical study.

There are still some limitations in our study, such as the weaknesses inherent in a retrospective study with lack of a systematic standardized follow-up and the inevitable losses to follow-up. The study failed to report any adverse events and further analysis of the potential mechanism on CHMs in treating XDRE infections is still needed. In addition, the therapeutic regimen was diverse in different cases. Whether it is the antibiotics therapy or the CHM therapy, each prescription was decided by the physician. Thus we can only perform a simple analysis on the efficacy evaluation. These issues weakened the strength of the study. However, this study is still a consequential study as it provides some preliminary support on the effectiveness of CHMs in treating XDRE infection based on the real-life data.

Based on this result reported so far, a large prospective study is required to better assess the effectiveness and safety of the applied CHMs in management of XDRE infection and identify further mechanism. To facilitate future trials, it is essential to have a reliable and validated CHM prescription instrument for XDRE infection; then a randomized, controlled, and double-blind clinical trial can be designed and developed based on this CHM prescription.

## 5. Conclusion

In our experience, CHMs combined with antibiotics therapy achieved more desirable effectiveness in treating XDRE infections compared with antibiotics monotherapy; CHMs might be a potential huge resource in the field of XDRE infection management and enlighten the new antibiotics research and development.

## Figures and Tables

**Table 1:** XDRE type and infected location distribution of included patients: total (TCM group/Control group).

Infection category	Total	Pulmonary infection	Urinary system infection	Postoperative wound infection
PsAr	190 (148/42)	156 (126/30)	35 (24/11)	19 (17/2)
AB	168 (100/68)	157 (91/66)	34 (18/16)	11 (9/2)
KP	136 (105/31)	116 (90/26)	18 (15/3)	9 (6/3)
E. coli	287 (205/82)	86 (61/25)	177 (136/41)	42 (32/10)
PsAr + AB	10 (7/3)	10 (7/3)	4 (3/1)	0
PsAr + KP	7 (7/0)	7 (7/0)	0	1 (1/0)
PsAr + E. coli	4 (4/0)	4 (4/0)	3 (3/0)	0
AB + KP	2 (2/0)	2 (2/0)	0	0
KP + E. coli	3 (2/1)	3 (2/1)	1 (1/0)	0
PsAr + AB + KP	1 (1/0)	1 (1/0)	0	0

PsAr: *Pseudomonas aeruginosa*; AB: *Acinetobacter baumannii*; KP: *Klebsiella pneumoniae*; E. coli: *Escherichia coli*. *Note*. The values in the table presented the total patient number who were infected with corresponding XDRE, followed by the patient number in TCM group (the former) and Control group (the latter) in the brackets. There might be more than 1 infected location in one patient; thus the total infected locations were not equal to the patient numbers.

**Table 2:** Baseline characteristics in TCM group and Control group (^*∗*^*P* < 0.05).

	TCM	Control	Normal value range
Case number	547	219	
Gender (M/F)	293/254	124/95	
Age (yr)			
Mean ± SD	68.01 ± 15.95	63.22 ± 18.73	
Range	18–101	18–98	
Temperature (°C)	36.77 ± 1.62	36.80 ± 2.38	36.5~37.5
Heart rate (/min)	83.92 ± 15.31	88.42 ± 18.29	60~100
Breath (/min)	19.84 ± 4.89^*∗*^	21.69 ± 15.23	16~20
WBC (10^∧^9/L)	10.29 ± 4.75	10.21 ± 5.03	4~10
N (%)	71.19 ± 14.71	74.35 ± 17.09	40~70
HGB (g/L)	103.65 ± 35.32^*∗*^	107.36 ± 29.42	120~160
RBC (10^∧^12/L)	3.77 ± 1.64	3.91 ± 2.46	3.5~5.5
PLT (10^∧^9/L)	218.65 ± 105.86	229.79 ± 110.74	100~300
Cr (*μ*mol/L)	81.76 ± 81.85	88.54 ± 93.18	53~106
GLU (mmol/L)	6.85 ± 2.75	7.22 ± 3.22	3.9~6.1
TBIL (*μ*mol/L)	15.65 ± 30.20^*∗*^	26.84 ± 50.27	2.0~20.4
ALB (g/L)	33.76 ± 7.08	33.85 ± 15.25	40.0~55.0

^*∗*^There was significant difference between the two groups (*P* < 0.05). There were no significant differences between the TCM and Control group (*P* > 0.05) concerning all items except breath, HGB, and TBIL.

**Table 3:** Change of each index on the 3rd, 5th, 7th, and 14th day after treatment in TCM group and Control group (^*∗*^*P* < 0.05 between the 2 groups).

	3 days after treatment		5 days after treatment		7 days after treatment		14 days after treatment	
	TCM	Control	TCM	Control	TCM	Control	TCM	Control
Temperature	36.78 ± 0.81	36.98 ± 0.78	36.76 ± 0.60^**∗**^	36.67 ± 2.65	36.71 ± 0.56^**∗**^	36.49 ± 0.49	36.60 ± 0.72	36.68 ± 1.64
Heart rate	82.72 ± 14.5^**∗**^	88.32 ± 18.72	82.09 ± 13.46^**∗**^	85.93 ± 17.34	82.08 ± 14.14	85.20 ± 15.96	81.85 ± 11.52	81.95 ± 13.94
Breath	19.53 ± 4.04^**∗**^	20.71 ± 6.15	19.29 ± 3.94^**∗**^	20.33 ± 6.19	19.45 ± 3.86	20.12 ± 5.76	18.61 ± 1.86^**∗**^	19.18 ± 4.01
WBC	9.48 ± 4.83	10.43 ± 5.37	9.18 ± 5.40^**∗**^	10.82 ± 9.42	8.12 ± 4.32^**∗**^	9.72 ± 6.77	8.26 ± 5.26	9.55 ± 9.71
N %	72.29 ± 15.45^**∗**^	77.49 ± 13.32	70.76 ± 15.23	74.32 ± 13.24	68.48 ± 14.24^**∗**^	73.24 ± 13.83	66.90 ± 14.04^**∗**^	72.57 ± 12.84
HGB	98.62 ± 31.97^**∗**^	105.57 ± 26.09	100.59 ± 32.72	105.42 ± 27.14	101.72 ± 59.43	103.31 ± 26.53	104.21 ± 29.9^**∗**^	102.13 ± 25.74
RBC	3.56 ± 1.08	3.59 ± 0.63	3.55 ± 0.81	3.75 ± 1.61	3.70 ± 1.73	3.69 ± 1.43	3.74 ± 2.15	3.90 ± 2.13
PLT	216.42 ± 97.12	215.65 ± 104.63	225.90 ± 117.62	236.07 ± 116.18	222.79 ± 110.07^**∗**^	243.46 ± 116.80	218.52 ± 98.37^**∗**^	250.54 ± 112.43
Cr	86.14 ± 82.93	93.13 ± 81.44	86.58 ± 95.03	79.59 ± 70.60	85.57 ± 85.74	83.33 ± 83.24	68.24 ± 44.68^**∗**^	75.84 ± 54.04
GLU	6.92 ± 2.54^**∗**^	7.51 ± 2.69	6.54 ± 2.04	6.71 ± 2.50	6.66 ± 2.16	6.67 ± 2.40	6.29 ± 1.97^**∗**^	6.56 ± 2.25
TBIL	13.77 ± 15.34^**∗**^	23.08 ± 30.90	12.61 ± 8.02^**∗**^	23.32 ± 38.24	16.58 ± 42.05^**∗**^	24.39 ± 50.82	13.67 ± 11.47^**∗**^	22.34 ± 60.05
ALB	31.89 ± 7.07	30.55 ± 6.02	32.32 ± 7.53	31.15 ± 6.56	33.54 ± 7.05^**∗**^	31.68 ± 5.85	33.04 ± 6.87^**∗**^	31.93 ± 6.44

^*∗*^There was significant difference between the TCM and Control groups (*P* < 0.05).

## Abbreviations

XDRE: Extensively drug-resistant enterobacteria
NFB: Nonfermentative bacteria
CHM: Chinese herbal medicine
TCM: Chinese medicine treatment
WBC: White blood cell
N%: Percentage of neutrophil
HBG: Hemoglobin
RBC: Red blood cell
PLT: Platelet
GLU: Blood glucose
Cr: Creatinine
BUN: Blood urea nitrogen
TBIL: Total bilirubin
ALB: Albumen
PsAr: * Pseudomonas aeruginosa*
AB: * Acinetobacter baumannii*
KP: * Klebsiella pneumoniae*
*E. coli*: * Escherichia coli*
ICU: Intensive care units
CDC: Centers for Disease Control and Prevention
ADR: Adverse drug reaction
SIRS: Systemic inflammatory response syndrome.
